# Mutation analysis of Chinese sporadic congenital sideroblastic anemia by targeted capture sequencing

**DOI:** 10.1186/s13045-015-0154-0

**Published:** 2015-05-20

**Authors:** Wenbin An, Jingliao Zhang, Lixian Chang, Yingchi Zhang, Yang Wan, Yuanyuan Ren, Deyun Niu, Jian Wu, Xiaofan Zhu, Ye Guo

**Affiliations:** Division of Pediatric Blood Diseases Center, Institute of Hematology and Blood Diseases Hospital, Chinese Academy of Medical Sciences & Peking Union Medical College, 288 Nanjing Road, Tianjin, 300020 People’s Republic of China; MyGenostics Inc., Baltimore, MD USA; State Key Laboratory of Experimental Hematology, Institute of Hematology and Blood Diseases Hospital, Chinese Academy of Medical Sciences & Peking Union Medical College, 288 Nanjing Road, Tianjin, 300020 People’s Republic of China

**Keywords:** Congenital sideroblastic anemia, Targeted capture sequencing, Molecular genetics, Clinical characteristics

## Abstract

**Background:**

Congenital sideroblastic anemias (CSAs) comprise a group of heterogenous genetic diseases that are caused by the mutation of various genes involved in heme biosynthesis, iron-sulfur cluster biogenesis, or mitochondrial solute transport or metabolism. However, approximately 40 % of patients with CSA have not been found to have pathogenic gene mutations. In this study, we systematically analyzed the mutation profile in 10 Chinese patients with sporadic CSA.

**Findings:**

We performed targeted deep sequencing analysis in ten patients with CSA using a panel of 417 genes that included known CSA-related genes. Mitochondrial genomes were analyzed using next-generation sequencing with a mitochondria enrichment kit and the HiSeq2000 sequencing platform. The results were confirmed by Sanger sequencing. The ALAS2 mutation was detected in one patient. SLC25A38 mutations were detected in three patients, including three novel mutations. Mitochondrial DNA deletions were detected in two patients. No disease-causing mutations were detected in four patients.

**Conclusion:**

To our knowledge, the pyridoxine-effective mutation C471Y of ALAS2, the compound heterozygous mutation W87X, I143Pfs146X, and the homozygous mutation R134C of SLC25A38 were found for the first time. Our findings add to the number of reported cases of this rare disease and to the CSA pathogenic mutation database. Our findings expand the phenotypic profile of mitochondrial DNA deletion mutations. This work also demonstrates the application of a congenital blood disease assay and targeted capture sequencing for the genetic screening analysis and diagnosis of heterogenous genetic CSA.

**Electronic supplementary material:**

The online version of this article (doi:10.1186/s13045-015-0154-0) contains supplementary material, which is available to authorized users.

## Findings

### Introduction

Sideroblastic anemias comprise a group of disorders that share several characteristics including mitochondrial iron accumulation in bone marrow erythroid precursors (ringed sideroblasts), ineffective erythropoiesis, increased levels of tissue iron, and varying proportions of hypochromic erythrocytes in the peripheral blood [1, 2]. Congenital sideroblastic anemias (CSAs) are rare diseases that are typically characterized by germline genetic mutations that cause defects in mitochondrial heme synthesis, iron-sulfur cluster metabolism, or protein synthesis [1, 2]. Recently, SF3B1 mutations were found in 70 % of patients with myelodysplastic syndrome with ringed sideroblast but were not detected in patients with CSA [3, 4]. The most common CSA is X-linked sideroblastic anemia (XLSA); this CSA is caused by mutations in ALAS2, which encodes 5-aminolevulinate synthase [2, 5]. Mutations in the erythroid-specific mitochondrial transporter SLC25A38 are the most common cause of autosomal recessive CSA [5, 6]. Other known etiologies include mutations in the genes for SLC19A2, GLRX5, PUS1, ABCB7, YARS2, and mitochondrial DNA deletions [5, 7–13]. However, approximately 40 % of CSAs, either alone or accompanied by a syndrome, have no known pathogenic gene mutations [5, 14]. Here, we used a congenital blood disease assay and targeted capture sequencing to genetically screen 10 Chinese patients with sporadic CSA.

### Patients and methods

The study protocol was approved by the Institutional Review Boards of the Hematology and Blood Diseases Hospital, CAMS/PUMC (Ethics No. KT2013004-EC-1). Informed consent was obtained from the guardians of the patients following institutional guidelines. We collected ten patients with CSA according to the diagnostic criteria referred to in previous reports [14]. Total DNA was extracted from the bone marrow or peripheral blood leukocytes and oral epithelial cells of the patients and from peripheral blood leukocytes of the available family members using standard methods. We designed a targeted capture sequencing assay to test a panel of 417 blood disease genes, including the seven known CSA-related genes. The targeted genes were enriched using a biotinylated capture probe (MyGenostics, Baltimore, MD, USA) as described previously [15]. Sanger sequencing was used to confirm the mutations. Mitochondrial genome capture sequencing was performed using a mitochondria enrichment kit (MitoCap™, MyGenostics, Beijing, China) as described previously [16]. The enrichment libraries were sequenced using an Illumina HiSeq 2000 sequencer. The 417 genes in the panel that was used for targeted capture sequencing are listed in Additional file 1: Table S1. The primers used for Sanger sequencing are listed in Additional file 2: Table S2. Detailed experimental methods are described in Additional file 3: Figure S1.

### Results

#### Mutation analysis

**Fig. 1:**
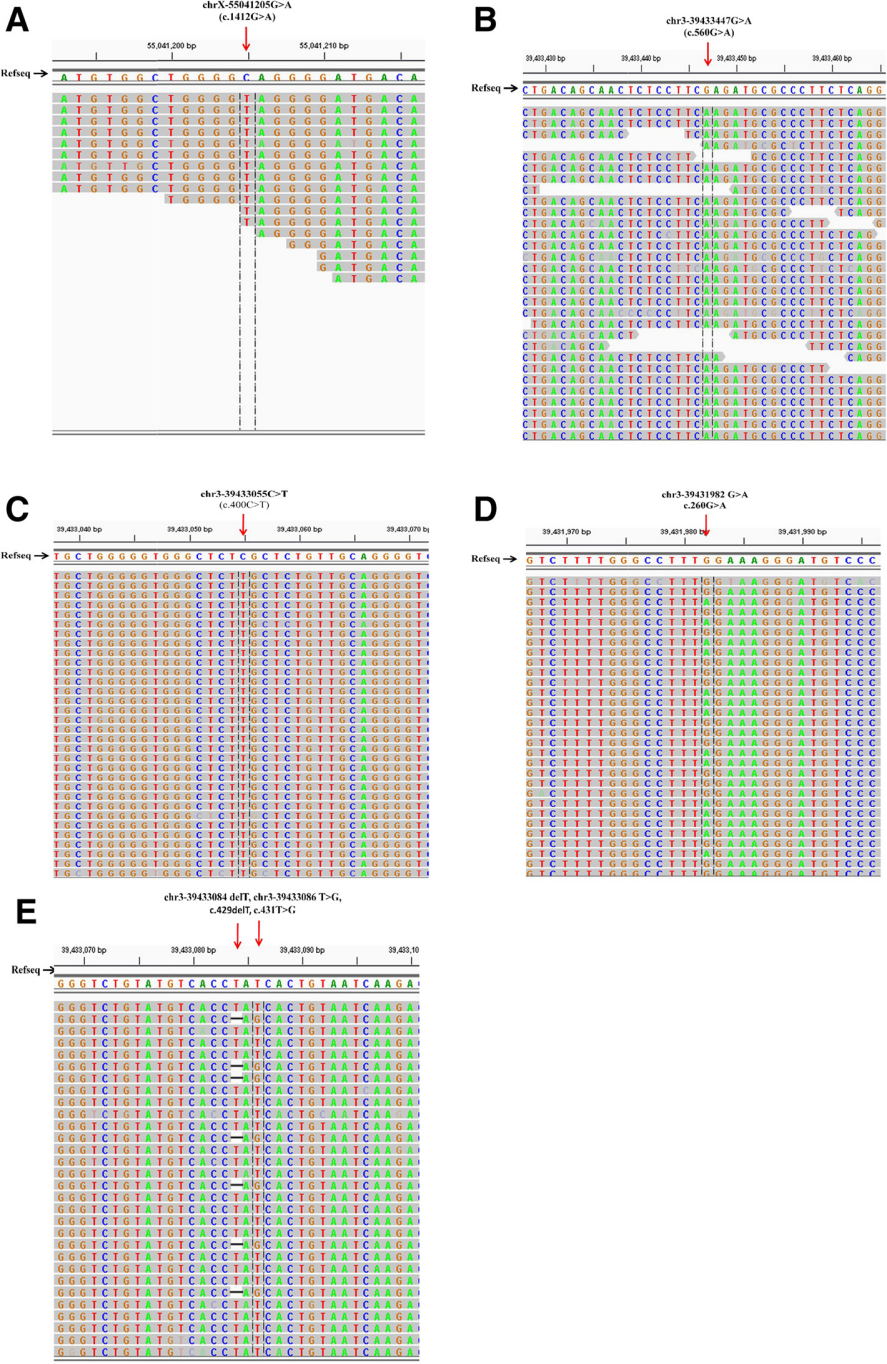
The results of mutations of ALAS2 and SLC25A38, which were detected in this study by targeted sequencing. **a** Homozygous missense mutation c.1412G > A of ALAS2 in Patient no. 3. **b** Homozygous missense mutation c.400C > T of SLC25A38 in Patient no. 4. **c** Homozygous missense mutation c.560G > A of SLC25A38 in Patient no. 5. **d** Heterozygous nonsense mutation c.260G > A of SLC25A38 in Patient no. 6. **e** Heterozygous nonsense mutations c.429delT and c.431 T > G of SLC25A38 in Patient no. 6

Among the ten patients, four exhibited nuclear genetic abnormality, and two exhibited mitochondrial genome deletions. One novel homozygous mutation, C471Y (c.1412G > A) of ALAS2, was detected in Patient no. 3. Five mutations in SLC25A38 were detected in three patients, including two homozygous mutations, R134C (c.400C > T) and R187Q (c.560G > A), and three heterozygous mutations, W87X (c.260G > A) and I143Pfs146X (c.429delT, c.431 T > G). Three of the mutations are novel, including R134C, W87X, and I143Pfs146X. Heterozygous mutation I143Pfs146X was detected in the mother of Patient no. 6, and heterozygous mutation W87X was detected in his father. Therefore, the mutations of Patient no. 6 were inherited from his father and mother, respectively, and caused compound heterozygous mutation in two alleles. The results of the mutation analysis are presented in Table 1, Fig. 1, and Additional file 4: Figure S2.

**Table 1:** Clinical and laboratory features of ten patients with CSA and results of mutation analyses

ID	Gender	ACD	HGB (g/L)	MCV (fL)	RDW-CV (%)	RET (%)	sFER (ng/mL)	TS (%)	RS (%)	Complications	Genetic mutation	hom/het	Result	Prognosis	Response to PPL
1	M	At birth	50	88.1	15.1	0.68	505.96	28	28	Diabetes mellitus	MtDNA 6250–12,498 del	-	COX1-ND5del	Normal HGB at 11-month-old	No
2	M	0.5 m	55	85.6	16.9	0.68	1101.9	ND	35	-	MtDNA 8647–14399del	-	ATP6-ND5del	Died at 7-month-old	No
3	M	14 y	20	62	20.3	ND	6020	84	24	-	ALAS2 c.1412G > A	hom	p.C471Y	Normal HGB after PPL treatment	Yes
4	M	2 m	50	68.8	34.5	2.06	450.6	90	32	-	SLC25A38 c.400C > T	hom	p.R134C	Transfusion dependence	No
5	F	5 m	52	74.2	25.4	0.72	197	91	55	Hypospadias	SLC25A38 c.560G > A	hom	p.R187Q	Loss to follow-up	No
6	M	3 m	49	74.4	ND	0.17	1434.5	81	64	-	SLC25A38 c.260G > A,c.429delT,c.431 T > G	het	p.W87X, p.I143Pfs146X	Transfusion dependence	No
7	M	5 y	65	69.9	34.1	0.35	1296.3	98	M	-	ND	-	-	Transfusion dependence	No
8	M	1 y	70	80.8	29.6	1.72	73.14	35	40	-	c.1997_1998insTAAT, c.2155_2156ins16	het	Frameshift mutation	Transfusion dependence	No
9	M	At birth	69	67	25.9	0.81	51.3	88	40	-	ND	-	-	Transfusion dependence	No
10	F	At birth	51	62.1	35.5	0.41	554.43	92	48	-	ND	-	-	Transfusion dependence	No

*M* male, *F* female, *ACD* age clinical detected, *m* months, *y* years, *HGB* hemoglobin, *MCV* Mean Corpuscular Volume, *RDW-CV* red cell distribution width, reference range 11 % to 14.1 %, *RET* reticulocyte count, *sFER* serum ferritin, *TS* transferrin saturation, *RS* ring sideroblast, *MtDNA* mitochondrial DNA, *ND* not detected, *hom* homozygous mutation, *het* heterozygous mutation, *PPL* pyridoxine

**Fig. 2:**
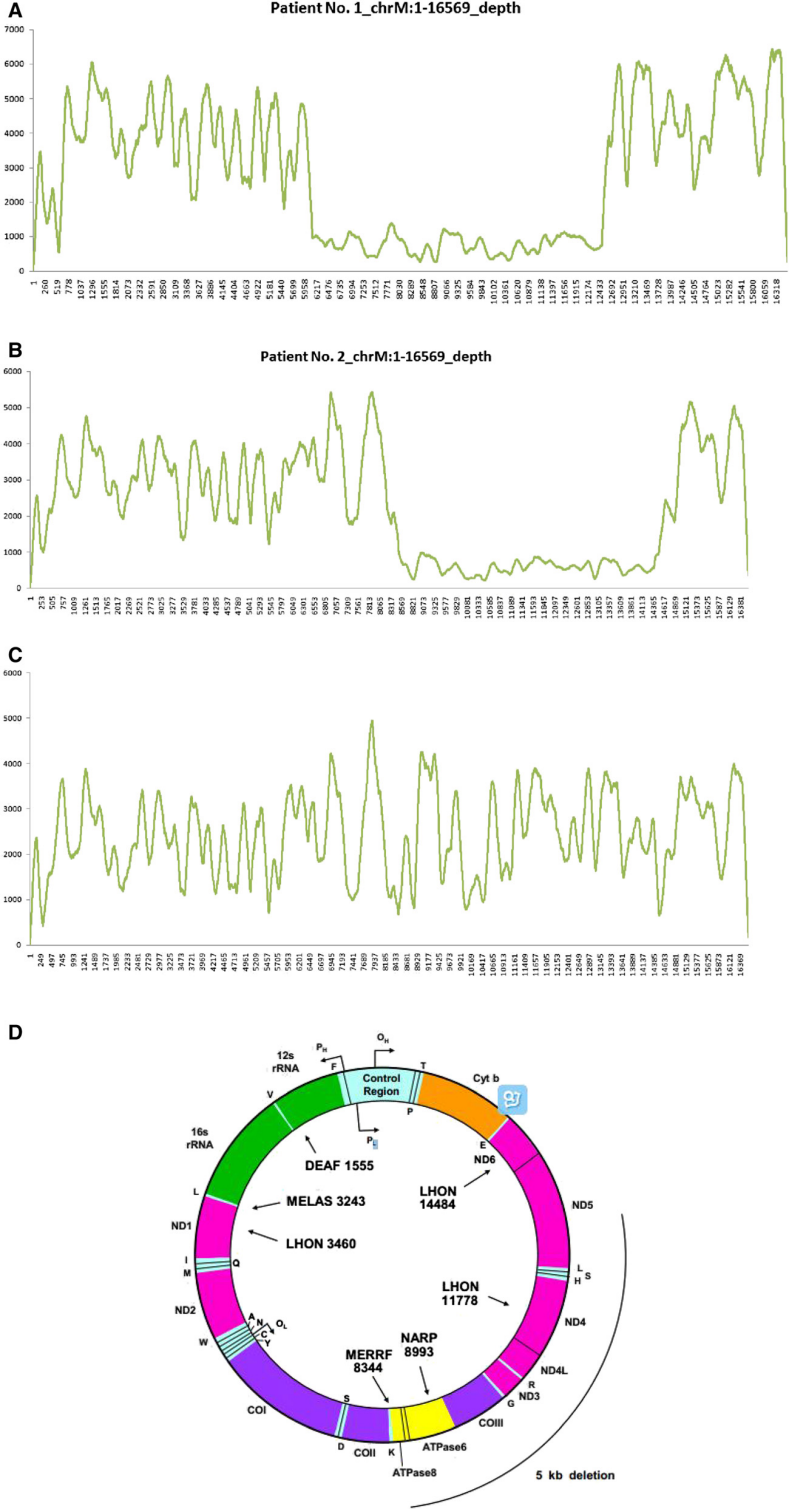
Mitochondrial DNA capture sequencing identified deletion mutations in two patients with Pearson marrow-pancreas syndrome. **a** Mitochondria coverage graph of Patient no. 1. **b** Mitochondria coverage graph of Patient no. 2. **c** Mitochondria coverage graph of the normal control. **d** The morbidity map of the human mtDNA genome shows that a deletion of range 6250–12,498 causes truncation of the mitochondrial genes COXI, COXII, ATP8, ATP6, COXIII, ND3, ND4L, ND4, and ND5 and of eight tRNA genes (S, D, K, G, R, LCUN_,_ SAGY, and H) in Patient no. 1. In addition, the deletion of m. 8647–14,399 causes truncation of the mitochondrial genes ATP6, COIII, ND3, ND4L, ND4, and ND5 and of five tRNA genes (G, R, LCUN, SAGY, and H) in Patient no. 2

Large mitochondrial genome deletions were detected in two patients thus confirming the diagnosis of Pearson marrow-pancreas syndrome (PMPS). Patient nos. 1 and 2 exhibited novel deletions of 6249 bp and 5753 bp, respectively, resulting in the deletion or truncation of mitochondrial genes. Details of the affected genes are presented in Fig. 2.

In the remaining four patients, no disease-causing mutations were detected. Mitochondrial genes involving shared deletion fragments are listed in Table 2.

**Table 2:** A list of mitochondrial genes involving shared deletion fragments

	Mitochondrial genes
8527..9207	ATP6 (partly delete)
9207..9990	COX3
9991..10058	TRNG tRNA-Gly
9991..10058	TRNG tRNA-Gly
10059..10404	ND3
10405..10469	TRNR tRNA-Arg
10470..10766	ND4L
10760..12137	ND4
12138..12206	TRNH tRNA-His
12207..12265	TRNS2 tRNA-Ser
12266..12336	TRNL2 tRNA-Leu
12337..14148	ND5 (partly delete)

#### Clinical features

Patient no. 3 exhibited typically microcytic-hypochromic anemia and responded well to pyridoxine treatment. Patients with mutations in SLC25A38 had severe microcytic-hypochromic anemia and systemic iron overload very early in life. The two patients with mitochondrial DNA deletions had severe macrocytic anemia at birth that was accompanied by various degrees of neutropenia and thrombocytopenia and exhibited different degrees of dysplasia in bone marrow cell morphology; in particular, vacuoles were present in myeloid and erythroid precursors. These patients developed severe iron overload shortly after birth. Additionally, Patient no. 1 exhibited congenital insulin-dependent diabetes mellitus. At the end of follow-up, the hemoglobin level of Patient no. 1 (11 months old) gradually increased to normal levels since the age of 6 months, and the Patient no. 2 died at the age of 7 months. The clinical features of the patients are listed in Table 1.

Patients without disease-causing mutations presented severe microcytic anemia and were dependent on transfusions since infancy or childhood. Additionally, the bone marrow histopathology and immunohistochemistry of Patient no. 8 exhibited abnormal lymphocyte infiltration, including 35 % of PAX5+, CD79a+, CD20+ B lymphocytes 10 % of CD10+, TdT+ B lymphocytes, and a small number of CD5 + T cells; the positive rate of Ki67 was 70 %, and Bcl-2 was negative. This phenotype was uncommon in previous reports. The ratio of lymphocytes in peripheral blood was normal. In addition, Patient no. 8 exhibited decreased levels of immunoglobulins (IgG 5.75 g/L [7.51–15.6], IgA 0.47 g/L [0.82–4.53], C3 0.67 g/L [0.79–1.52]). Immune fixation electrophoresis demonstrated the absence of monoclonal immunoglobulins and light chains. The patient did not respond to pyridoxine. Recently, mutations in TRNT1 were detected in patients with syndromic CSA that was associated with B cell immunodeficiency, periodic fevers, and developmental delay (SIFD) [17, 18]. However, the patient in our study did not exhibit signs of immunodeficiency or developmental delay. Although two heterozygous frameshift mutations (c.1997_1998insTAAT, c.2155_2156insTATAAGAGATTTCTAA) in the same allele of TRNT1 were detected in Patient no. 8 by Sanger sequencing, the same mutations were detected in the father of Patient No. 8 thus leading us to speculate that these mutations represented a low frequency of polymorphisms. The major clinical features of these patients are listed in Table 1.

### Discussion

The ALAS2 gene is located on Xp11.21 and catalyzes the first and rate-limiting step in the heme biosynthetic pathway in erythroid cells. All of the disease-causing mutations affect the catalytic domain of ALAS2 (encoded by exons 5 to 11) or the enhancer region in intron 1 [5, 14, 19]. Typically, patients with XLSA that is related to the ALAS2 mutation responded to pyridoxine. The novel mutation ALAS2 C471Y observed here further expands the profile of pyridoxine-effective mutations that are recognized to cause XLSA. The mutated amino acid may be located in the middle of a highly conserved hydrophobic region of ALAS2, and its side chain may help to stabilize the PLP-binding site. Therefore, patients with this mutation may respond well to pyridoxine [20, 21].

Patients with CSA caused by SLC25A38 mutation commonly exhibit early onset, no gender differences, severe microcytic-hypochromic anemia, and are nonsyndromic [5, 6]. SLC25A38 might participate in the glycine transport within the mitochondrial membrane and/or the mutual transport of glycine with 5-aminolevulinic acid (ALA) through the mitochondrial membrane (promoting ALA synthesis). The homozygous mutations R134C and R187Q found here are consistent with previous findings of R134H and R187P/Q mutations [6]. R187 residue is the conserved arginine of the arginine-aspartate (RD) dipeptide of transmembrane helix 4, which is thought to provide contact points that determine substrate specificity [22]. In addition, R134 is close to R187 in the three-dimensional structure and is conserved among multiple species. The compound heterozygote mutations that were seen in Patient No. 6 cause protein truncation in both alleles and completely destroy the function of the protein completely and might have caused his far more serious anemia. The potential model of three-dimensional structures of human ALAS2 and its evolutionary conservation of the residues with missense mutations are showed in Additional file 5: Figure S3.

PMPS is associated with large-scale mitochondrial DNA (mtDNA) deletions, rearrangements, or duplications. Typical clinical manifestations include macrocytic anemia and symptoms of mitochondrial diseases with vacuolization of erythroid and myeloid precursors and ring sideroblasts [13]. The patients in our study exhibited typical hematological features but no symptoms of mitochondrial diseases. The deletion fragments were commonly in the sequence range 6250–12,498. The genes involved are listed in Table 2. Among these genes, the *COX3* gene product participates in iron ion oxidoreduction reactions as one of the cytochrome C oxidase subunits. Mutations in COX I (also a subunit of cytochrome C oxidase) have been confirmed in patients with acquired idiopathic sideroblastic anemia [23]. Therefore, we speculated that deletion of the *COX3* gene might affect the mitochondrial metabolism of iron in the same way. Mitochondrial tRNA participates in the synthesis of all 13 subgroups of enzymes involved in the oxidative phosphorylation of the respiratory chain. Among these proteins, the TRNG tRNA-Gly protein plays a role in mitochondrial glycine transport. Therefore, the deletion of this fragment might affect mitochondrial heme synthesis. ND3, ND4L, ND4, and ND5 are components of NADH dehydrogenase, which is the core element of iron-sulfur clusters. A lack of this enzyme might affect mitochondrial iron metabolism [24, 25]. In conclusion, deletions of mitochondrial genes might reduce heme synthesis and cause iron accumulation in the mitochondria by affecting the transport of cytochrome oxidase C, iron-sulfur clusters, and the substrate.

### Conclusion

In this study, we used targeted capture sequencing to detect etiologic mutations in a heterogeneous genetic disease. A novel pyridoxine-effective mutation of ALAS2 and a novel compound heterozygous mutation of SLC25A38 were identified. In addition, we identified fragmental deletions of MtDNA in two patients with CSA using mitochondrial genome capture sequencing, which provided clues for further research on iron metabolism. The utility of targeted capturing sequencing is clear for practical clinical use, especially for heterogenous genetic diseases.

## Additional files

Additional file 1: Table S1. A list of 417 blood disease-related genes in the panel for targeted capture sequencing. Additional file 2: Table S2. The primers that were used for sequencing CSA-related genes. Additional file 3: Figure S1. Detailed methods relating to DNA library preparation, targeted gene enrichment, and clustering, sequencing and bioinformatics analyses. Additional file 4: Figure S2. Chromatograms of the mutations found in the patients, as confirmed by Sanger sequencing. Additional file 5: Figure S3 Potential model of three-dimensional structure of human ALAS2 (A) and SLC25A38 (B) and their evolutionary conservation of the residues with missense mutations. The color scale range from blue to red represent the conservation scores from 1-most variable to 9-most conserved. The color code for residues with missense mutations C471 (A), R134 (B) are 6, 9, respectively. Surface-mapping of phylogenetic information was done with ConSurf; view is from the cytosolic side.
