# Supplementary material for: Mutation analysis of Chinese sporadic congenital sideroblastic anemia by targeted capture sequencing
Source: J Hematol Oncol. 2015 May 20;8:55. doi: 10.1186/s13045-015-0154-0 (PMC4490691; doi:10.1186/s13045-015-0154-0)
Supplement: Additional file 1: Table S1. — A list of 417 blood disease-related genes in the panel for targeted capture sequencing. [file 13045_2015_154_MOESM1_ESM.docx]

Additional file 1. A list of 417 blood disease-related genes in the panel for targeted capture sequencing.

| ABCA1 | CSF3R | FKHRL1 | KANSL1 | PHF9 | SNRNP200 |
| --- | --- | --- | --- | --- | --- |
| ABCB7 | CSMD1 | FLI1 | KDM6A | PIEZO1 | SOX2 |
| ABCC6 | CST3 | FLT3 | KIT | PIGA | SPTA1 |
| ABCC8 | CTC1 | FLT4 | KIT | PKLR | SPTB |
| ABCG5 | CTCF | FLVCR2 | KLF1 | PLG | SRP72 |
| ABCG8 | CUX1 | FOXC2 | KLF4 | POT1 | SRSF2 |
| ACTN1 | CXCR4 | FOXO3a | KLHDC8B | PPOX | STAG2 |
| ADA | CYB5R3 | FTL | KLKB1 | PRF1 | STAT3 |
| ADAMTS13 | CYCS | FTMT | KNG1 | PROC | STIM1 |
| ADD1 | CYP2A6 | FXN | KRAS | PROS1 | STN1 |
| AEBP2 | CYP2C9 | FXYD2 | KRG2 | PRPF40B | STX11 |
| AK1 | CYP4F2 | G6PC3 | KRIT1 | PRPF8 | STXBP2 |
| AK2 | DAPK1 | G6PD | LAMA3 | PTPMT1 | SUZ12 |
| ALAD | DAXX | GAR1 | LAMB4 | PTPN11 | TAT |
| ALAS2 | DCAF7 | GAS1 | LBR | PUS1 | TAZ |
| ALDH2 | DCLRE1B | GATA1 | LDLR | Rab27a | TBCE |
| ALDH4A1 | DCLRE1C | GATA2 | LIG4 | RAD21 | TBXAS1 |
| ALDM | DDX11 | GCLC | LIPI | RAD50 | TCAB1 |
| ALDOA | DDX41 | GFI1 | LMAN1 | RAD51C | TEN1 |
| ALG12 | Dido1 | GGCX | LMBRD1 | RAG1 | TERC |
| ALS2 | DIS3 | GK | LPL | RAG2 | TERF1 |
| ANK1 | DKC1 | GKLF | LUC7L2 | RAP1 | TERF2 |
| AP3B1 | DNAH2 | GLRX5 | LYRM4 | RARA | TERF2IP |
| APOA1 | DNAH7 | GNAS | LYST | RB1 | TERT |
| APOA5 | DNMT3A | GP1BA | MAP3K4 | RIT1 | TET2 |
| APOB | DST | GP1BB | MASTL | RNF55 | TF |
| ARG1 | EED | GP9 | MCFD2 | RPL11 | THBD |
| ARSA | EGF | GPI | MDM2 | RPL26 | THPO |
| ASXL1 | EGLN1 | GPRC5A | MDS1 | RPL31 | TIN2 |
| ATAD3B | ELANE | GPX1 | MEFV | RPL35a | TINF2 |
| ATM | ENO1 | GSR | MHF1 | RPL5 | TMPRSS6 |
| ATPIF1 | EPAS1 | GSS | MHF2 | RPS10 | TNFA |
| ATRX | EPB41 | HAMP | MLH1 | RPS14 | TNFRSF1A |
| B2M | EPB42 | HAX1 | MMACHC | RPS17 | TNFSF2 |
| B4GALT1 | EPOR | HBA1 | MMADHC | RPS19 | TP53 |
| BACH1 | ESCO2 | HBA2 | Mpl | RPS24 | TPI1 |
| BCAM | ETNK1 | HBB | MSH2 | RPS26 | TPP1 |
| BCOR | ETV1 | HBG1 | MTTP | RPS29 | TRF1 |
| BCORL1 | ETV6 | HBG2 | MYC | RPS37 | TRF2 |
| BLM | EZF | HCFC2 | MYH9 | RPS7 | TRPM6 |
| BMPR1A | EZH2 | HFE | NAGA | RTEL1 | TUBB1 |
| BOD1L | F11 | HFE2 | NBN | RUNX1 | U2AF1 |
| BRAF | F12 | HIF1A | NBS1 | S100A9 | U2AF2 |
| BRCA2 | F13A1 | HK1 | NCOR2 | SBDS | UBB |
| BRCC3 | F13B | HMBS | NF1 | SCN4A | UMODL1 |
| BRIP1 | F5 | HOX2F | NFKBIA | SEC23B | UNC13D |
| BTBD12 | F8 | HOXB4 | NFS1 | SEC23B | UNC18B |
| BTK | F9 | HPD | NHP2 | SERPINC1 | USP42 |
| BUB1B | FAAP100 | HRG | NOLA2 | SERPINE1 | UTX |
| C16orf57 | FAAP24 | HSCB | NOLA3 | SERPINF2 | VANGL2 |
| CALR | FAM109A | HSPA9 | NOP10 | SERPING1 | VHL |
| CASP10 | FAM5C | IDH1 | NOTCH3 | SETBP1 | VKORC1 |
| CBL | FANCA | IDH2 | NPM1 | SF1 | VPS13B |
| CD36 | FANCB | IKAROS | NR3C2 | SF3A1 | VPS45 |
| CDAN1 | FANCC | IKBKG | NRAS | SF3b1 | VWF |
| CDH23 | FANCD1 | IKZF1 | NT5C3 | SH2B3 | WAS |
| CEBPA | FANCD2 | IL6 | OCT4 | SH2D1A | WRAP53 |
| CETP | FANCE | IRF1 | ORAI1 | SLC11A2 | WT1 |
| CFH | FANCF | ISCA1 | OTC | SLC19A2 | XIAP |
| CFHR1 | FANCG | ISCA2 | OTF3 | SLC25A37 | XRCC2 |
| CFHR3 | FANCI | ISCU | P2RY12 | SLC25A38 | XRCC3 |
| CFI | FANCJ | ITGA2B | PALB2 | SLC37A4 | YARS2 |
| CFP | FANCL | ITGA2B | PANK2 | SLC40A1 | ZNF43 |
| CHL1 | FANCM | ITGB3 | PCCA | SLC4A1 | ZNF93 |
| CHLR1 | FANCN | ITK | PCCB | SLX4 | ZNRF4 |
| CKS2 | FANCQ | ITPKC | PDE4D | SMAD4 | ZRSR2 |
| CLDN16 | FAS | JAK1 | PFKM | SMARCAL1 | ZSWIM4 |
| CLDN19 | FBXW7 | JAK2 | PGD | SMC1A |  |
| CPN1 | FECH | JAK3 | PGK1 | SMC3 |  |
| CREBBP | FGA | JARID2 | PHF6 | SND1 |  |
