# Supplementary material for: Mutation analysis of Chinese sporadic congenital sideroblastic anemia by targeted capture sequencing
Source: J Hematol Oncol. 2015 May 20;8:55. doi: 10.1186/s13045-015-0154-0 (PMC4490691; doi:10.1186/s13045-015-0154-0)
Supplement: Additional file 2: Table S2. — The primers that were used for sequencing CSA-related genes. [file 13045_2015_154_MOESM2_ESM.docx]

Additional file 2. The primers that were used for sequencing CSA-related genes.

| Primer | Forward (5'to3') | Reverse (5'to3') |
| --- | --- | --- |
| ALAS2-1 | GAGGTGGTTTTGCTCAAGGTAT | GAGAGATACAAAGCCGAGGA |
| ALAS2-2 | TTCGTTACTTTCTTTTGTTAGTTTTC | TCTCTTCAGGAGTTGGAAACATCA |
| ALAS2-3 | GAAGAAAGTGGAAGAGGAATGTAGT | CCTCTGCTGCACTAACATAGGC |
| ALAS2-4 | GGCAAGCATTATCACACATTAGG | AATAACAAGGACAATCTGCATCA |
| ALAS2-5 | TGACTTCCCTTGGCTGTACGTTC | TCTCTTGTTCTGTTACTCCTTTCCTG |
| ALAS2-6 | TGACTAAAATGCTTCCTTGATTCA | GAATCACGTCTGAGCATCTGGC |
| ALAS2-7 | TTGCCAGTATTGATGTTAGGGGA | TGGAGGGACTAAATGAAACAATC |
| ALAS2-8 | GGGAAGTGATTTGAACAAGAACATC | GCAGGGGGATGAGGGTTAAGA |
| ALAS2-9 | TCCCATTGTACTGTCCCTTATCT | GGACTAGGAGAAAAGCCAGAAGA |
| ALAS2-10 | CTAGACTCATTGATCCCTCCTCA | AAGGAGCTTGAGGTCACATGTAT |
| ALAS2-11 | GCCTCTGCCTGTTTCACTAATC | AAACTTGCTGCCTCCGAGA |
| ALAS2-12 | CCTCCAGTTAGAACTGTTTCCTTC | GGCAGAAGACAGATTCTGGGTAT |
| ALAS2-13 | GAGGAGGTAAGAAGAGGCTGCTAG | TCTGACAGGAGCAAAGGCAC |
| ALAS2-14 | AGTCTCACTGTCATGTCCCTCAC | CTTCTGCACAATCTTGCTCTTC |
| ALAS2-15 | CTGAGATAGGGCTTTAGGGGTAC | TCTTGCTGAAATGTGGGACCCCA |
| ALAS2-16 | GCTGAAGCACACCATACCTGAC | CTTCTATTTATCCCCCCGAATG |
| ALAS2-17 | TCTACTTTCTAAATGCAGGACCCAG | CTAGATCCAGCAAGTGCCAGCAT |
| ALAS2-18 | GGTGAGTTTGCTGAGGTGGA | TGAACAAAACAGCATAACGAACTC |
| ALAS2-19 | TAAGGAGGGAAATTTAAGTCATGC | GAAGCAACCAAATAGAGGAGTAGC |
| ALAS2-20 | TCAACAATATACTGTGGAAATGCTT | GGAGGTGCAAACTGGTAACATC |
| ALAS2-21 | TACATTTTGTCAATTAGCCCTCC | CATCAAGTAAGTAACTCAGAGCC |
| ALAS2-22 | CATGATTTCTGTCTCACGTAAAGAG | TTGAACAAGTCATGAATCTTCTTTC |
| ALAS2-23 | GGAAAGAACTTACATGTACTAGGC | GATCTTGTCCCTGAAAAACTGGT |
| ALAS2-24 | ATGGGTAAAATACAGACTAGCCAGG | GTAGTTTGGAAGACCTAGAGGAAGT |
| ALAS2-25 | AAGGATGGGAGACCAAGCAAGG | GTGGATAAGAAGGAAGAACAATGGG |
| ALAS2-26 | TGGTATCCTGCAATACAGCATC | ATAAACTTCGAGAACTGACCAGG |
| ALAS2-27 | CACTATATCAATTCCGGGCTCATAC | AAATGATGTTGGATCAGGCTAGGAT |
| ALAS2-28 | GTCCACCCATAAGCCCCAACTAAAC | AGGTGGGCATGTGAAATTGGTAGAG |
| ALAS2-29 | TTGTCTTTCAAGCCATTTTCTCCAG | AAGACCTTGCTCTGCTTCTTCCTTC |
| ALAS2-30 | TCTTCCACCCCAAGGCAGTCAG | CTTTGGCATTTACTAGCTGTGTGACC |
| ALAS2-31 | TCTGAACAATTCTATGTGATAGGTG | CTTCCCTTAAATAAATCCATAAGAG |
| ALAS2-32 | CTCCTACCTCCTTTCTTTGCTCAAT | CTGGGATCAAGGCTTGACTCTTTGT |
| ALAS2-33 | CTCTGCCCAAACTGTTGATCCTTAG | TTCTGTCCAAACAACCCCTATAGCT |
| ALAS2-34 | GCATAAGATTGACATCATCTCTGG | GAGCAGCCGCACAGATTCTAG |
| ALAS2-35 | GTGACTTGGTGGACATGGT | CCCTTAGTATGTGATGTACTCC |
| ALAS2-36 | TGGATCAAGACAATTAATGGGTCAC | GGGATGGTTCTGGGTGAGAAGATAC |
| ALAS2-37 | GCTCTTTAGACTAACCTAGTATTTC | TAAACTCAGTGGTCTCTGTCCT |
| ALAS2-38 | AGCCCTCAGATGATGGAAGATT | CAGAAATCCAACAAACATTAGGT |
| ALAS2-39 | ATGTAGGGTCATAGGAATGGAGTGG | CTCTGCTAGACCATCAACTGCCTAC |
| ALAS2-40 | ACCATGCCTAGCTAATTTCTGTATT | AGGTTGTTGTGGGGATTAGATG |
| ALAS2-41 | AGAGATAGGAAAGCAGTGGGTATGT | TAGATAACTTTTGCAGGATTTCAGG |
| ALAS2-42 | TTGTTCAGTATTCTCTTTTGCTCTG | CTCTGTCCTATATGTCAATGTTTCC |
| ALAS2-43 | TCAAGCATCACTGATACTGATCTTT | CTTGCCACTCTAATACTACAACCCT |
| ALAS2-44 | GCTTACATTCTAGTGGAAGGGGTAG | ATCCCATCTATCAGCAAAGACCCTT |
| ALAS2-45 | GGTGGAAGCAGGGAGACCCAAGAGT | TCATTGGTGCTATGACCAGGAAACC |
| ALAS2-46 | AGTCATGTGACTAGACAAGATC | AATGGGTTAGACTAGATCTTC |
| ALAS2-47 | ATCCCAGCATCCAGATACAAAT | GCATTTCCTTCCTCTTTCTCAT |
| SLC25A38 exon1 | GTCGTCCACGCTGGTCTCCA | CCCCGGCAATTCCGCCCTTT |
| SLC25A38 exon2 | TGAGGCACCACCAGGTAAGTGT | GCTGCTCAGGAACGGACCCC |
| SLC25A38 exon3 | AGGAAGTGTTTGAGTGGGGAATTGTTT | AGACCACATAGGTACTCCCACCACT |
| SLC25A38 exon4 | TGGGGTCTTTTGGGAAAACCCAGC | GTGACTCGCATGGAGGCGCT |
| SLC25A38 exon5 | GCCCCATAACCTGCAGTCTGCTT | CACCCTATCCTCACCCCGCCA |
| SLC25A38 exon6 | GGTGGGCAACTTGCACTGACCT | GCCTAGATTTTAACCTGGGCATGGGG |
| SLC25A38 exon7 | ACCCTCACTGTGGTACCAAG | CCTGGTTTTCCAGGTAGGAC |
| ABCB7 exon1 | ACAGCTGAAGCCTCCTCCCAGG | CCCCGAGGTCAGGAGGGCAA |
| ABCB7 exon2 | TGATCCGCCCGCCTTAGCCT | TCTCTGCATTTCCAGAAGCAGAAACAT |
| ABCB7 exon3 | AGTGAATGACACTGGGAAAGCCAG | ACCTTGAAGCACACGCACACACA |
| ABCB7 exon4 | ACCAAGCCCTCTGCTTTCCTAAAAGG | AGTGATTTACACCAGGCCCAGGA |
| ABCB7 exon5 | AGCCTGAAATGACAGCTCTCCCA | AACCTCCTTGAAGAAAGTCAACACCTG |
| ABCB7 exon6 | TCCACAGTAATGCCATGTGGGCT | CCCATGGGCATGCAACAGTACA |
| ABCB7 exon7 and 8 | CACGTACATAACTTCACGCCACCA | GGGACCAACATCATAGATGCCAAAACA |
| ABCB7 exon9 | TCAGGGGAAGGCTTTGTGAAGGA | CCAATCAGTGAGTGAGGCAGTGCT |
| ABCB7 exon10 | GGTGGGTCTTTCCCATTCCTAACG | AGCACCCCCACCCCTGACAA |
| ABCB7 exon11 and 12 | CCCTCCCCAACCCCACCTCA | GAGGCCCCAGGCCACACAAC |
| ABCB7 exon13 | ACCCCTGGGAAGGGAATGGGA | ACCCAATCAAATGTGACTCAACGAGCA |
| ABCB7 exon14 | GCCTCATTCTCATTCTTCCCACCTGC | TGGAAAAAGGGGGATAGGCATTTTGCT |
| ABCB7 exon15 | AGTTGCCTTCTCTTTTTGCTTTCTCCT | AGGGGCTAAAAACAGAATCGTAACAGG |
| ABCB7 exon16 | GGCACTGGGTAGCTCAACAGGGA | TGAGCACAACCAGGACAGTGACA |
| GLRX5 exon1 | CCGCGCCTCTCCCAGTTGTCT | CCGGCTCGAACCTTCAGACAGAC |
| GLRX5 exon2 | GGGAAGCCAGGGAGGGACAGTG | CAGGGCTCCAGAGATAGGCAGGTG |
| SLC19A2 exon1 | CAATGGAAGAGCAGGCAAGT | CGCTTTTCTCGGTCCTCTCT |
| SLC19A2 exon2 | CCAGGTCCTTTCATCACTAATGT | GCCCCCATAGTAGCAATTACA |
| SLC19A2 exon3 | TGGGCCTGTAAATTGCTTTC | CAAATTTGGGAGGGGTGAAT |
| SLC19A2 exon4 | GCAACAGCATTTGTGTAGCA | ACAATGCTTCCTCCCATTTG |
| SLC19A2 exon5 | CATTTGGTTGGAAAGGCAAT | TCACCCTGATCAAGTCACACA |
| SLC19A2 exon6 | GGCACGTGGTGTAAGTATGC | TGCTGTGCAGAGTTCTTGCT |
| PUS1 exon 1 | CTGCCTCTTCTCCCGAATTA | GTTCTCCGTCCTCCCAGAC |
| PUS1 exon 2 | GCGTCCGTCCTGGTAATG | CTTGGGCCTGCACCTAAAT |
| PUS1 exon 3 | AACATCATGGCGACCTCTGT | AACAAGCCAGAACACTGAGC |
| PUS1 exon 4 | ATGACGTAAGGTCCGCGTAG | GACAGTTGGAAAAGGCAGGA |
| PUS1 exon 5 | CATCCAGGCACTTCTCACCT | TGCGGGGAAGTGACACAC |
| PUS1 exon 6 | ATCCAGGAGCAGTGGAGAGA | CTCCTGGGCCTTTCTCTTTT |
| TRNT1 exon 1 | AGCTGCCAGTTGTCTCAGAT | GCTATTTTGCCCACTGCTGA |
| TRNT1 exon 2 | ATCCCTCAAACTGACACCGT | GGCTTAAAAAGTTAACCCTC |
| TRNT1 exon 3 | AAGCCTCAGTTAAATGAATC | GACGGGCATAGAAGAGACT |
| TRNT1 exon 4 | TTTAGGTATGGTAGTTGACG | CAATCTGCTAAACCATCT |
| TRNT1 exon 5 | GGCTCCACTCCACCTCATA | AAAGTATATGTGCGTTCTG |
| TRNT1 exon 6 | GGAGAAATGACCAGTTGCTT | CTCTTCCCTACTCACGACAA |
| TRNT1 exon 7 | TCGCCTTTAAGTTGGAACAT | AACATTTAGTTTCGTCACAC |
| TRNT1 exon 8-1 | TATATACTAGGCTTGGTCAG | TTATGCAAACTTACTTGGTC |
| TRNT1 exon 8-2 | TCTATCTTAACCTGTTCAG | GAGGCTTTCTTGCATCATAG |

PCR amplification were done in a total volume of 25 ml PCR mix containing at least 50 ng template DNA, using quick Taq PCR

^TM^ HS Dye mix (Qiagen) under the following conditions: 95°C for 3min, 35 cycles of 94°C for 30 sec, 61°C for 30 sec, and 72°C for 30

sec, one cycle of 72°C for 7min. All PCR products were confirmed by 2% agarose gel electrophoresis, purified using QIA quick PCR purification kit (Qiagen, Santa Clarita, CA) and directly bidirectional sequenced on a DNA sequencer using ABI PRISM 3730×l DNA Analyzer (Applied Biosystems, Foster City, CA). The sequence data files were analyzed using the Mutation Surveyor 3.25 software; average quality score was 20 or greater. All mutations were compared with published SNP data (dbSNP; [http://www.ncbi.nlm.nih.gov/projects/SNP](http://www.sciencedirect.com/science?_ob=RedirectURL&_method=externObjLink&_locator=url&_cdi=271220&_issn=01452126&_origin=article&_zone=art_page&_plusSign=%2B&_targetURL=http%253A%252F%252Fwww.ncbi.nlm.nih.gov%252Fprojects%252FSNP)) and cloned by the TA cloning system (Tiangen Biotech, Peking, China), with the PCR products spanning the original mutation points. A minimum of 10 wild-type clones per subject were considered to exclude a mutation.
