# Supplementary material for: Mutation analysis of Chinese sporadic congenital sideroblastic anemia by targeted capture sequencing
Source: J Hematol Oncol. 2015 May 20;8:55. doi: 10.1186/s13045-015-0154-0 (PMC4490691; doi:10.1186/s13045-015-0154-0)
Supplement: Additional file 3: Figure S1. — Detailed methods relating to DNA library preparation, targeted gene enrichment, and clustering, sequencing and bioinformatics analyses. [file 13045_2015_154_MOESM3_ESM.docx]

Additional file 3. Detailed methods relating to DNA library preparation, targeted gene enrichment, and clustering, sequencing and bioinformatics analyses.

**DNA Library Preparation**

Each DNA sample is quantiﬁed by agarose gel electrophoresis and Nanodrop (Thermo). Libraries were prepared using Illumina standard protocol. Briefly, 3 microgram of genomic DNA was fragmented by nebulization, the fragmented DNA is repaired, an ‘A’ is ligated to the 3′ end, Illumina adapters are then ligated to the fragments, and the sample is size selected aiming for a 350–400 base pair product. The size selected product is PCR amplified (Each sample is tagged with a unique index during this procedure), and the final product is validated using the Agilent Bioanalyzer.

**Targeted genes enrichment**

The amplified DNA was captured with a blood-disease related Gene Panel or Mitochondria genome using biotinylated oligo-probes (MyGenostics GenCap Enrichment technologies). The probes were designed to tile along 417 blood-disease related genes or the 16569bp mitochondria genome. The capture experiment was conducted according to manufacturer’s protocol. In brief, 1μg DNA library was mixed with Buffer BL and GenCap gene panel probe (MyGenostics, MD, USA), heated at 95°C for 7 min and 65°C for 2 min on a PCR machine; 23μl of the 65°C prewarmed Buffer HY (MyGenostics, MD, USA) was then added to the mix, and the mixture was held at 65°C with PCR lid heat on for 22 hours for hybridization. 50 μl MyOne beads (Life Technologies Corporation, USA) was washed in 500μL 1X binding buffer for 3 times and resuspended in 80μl 1X binding buffer. 64 μl 2X binding buffer was added to the hybrid mix, and transferred to the tube with 80μl MyOne beads. The mix was rotated for 1 hour on a rotator. The beads were then washed with WB1 buffer at room temperature for 15 minutes once and WB3 buffer at 65°C for 15 minutes three times. The bound DNA was then eluted with Buffer Elute. The eluted DNA was finally amplified for 15 cycles using the following program: 98℃ for 30 s (1 cycle); 98℃ for 25 s, 65℃ for 30 s, 72 ℃ for 30 s (15 cycles); 72 ℃ for 5 min (1 cycle). The PCR product was purified using SPRI beads (Beckman Coulter, Inc.) according to manufacturer’s protocol. The enrichment libraries were sequenced on Illumina HiSeq 2000 sequencer for paired read 100bp.

**Clustering and Sequencing**

Illumina utilizes a unique "bridged" amplification reaction that occurs on the surface of the flow cell. A flow cell containing millions of unique clusters is loaded into the HiSeq 2000 for automated cycles of extension and imaging. Illumina's Sequencing-by-Synthesis utilizes four proprietary nucleotides possessing reversible fluorophore and termination properties. Each sequencing cycle occurs in the presence of all four nucleotides leading to higher accuracy than methods where only one nucleotide is present in the reaction mix at a time. This cycle is repeated, one base at a time,generating a series of images each representing a single base extension at a specific cluster.

***
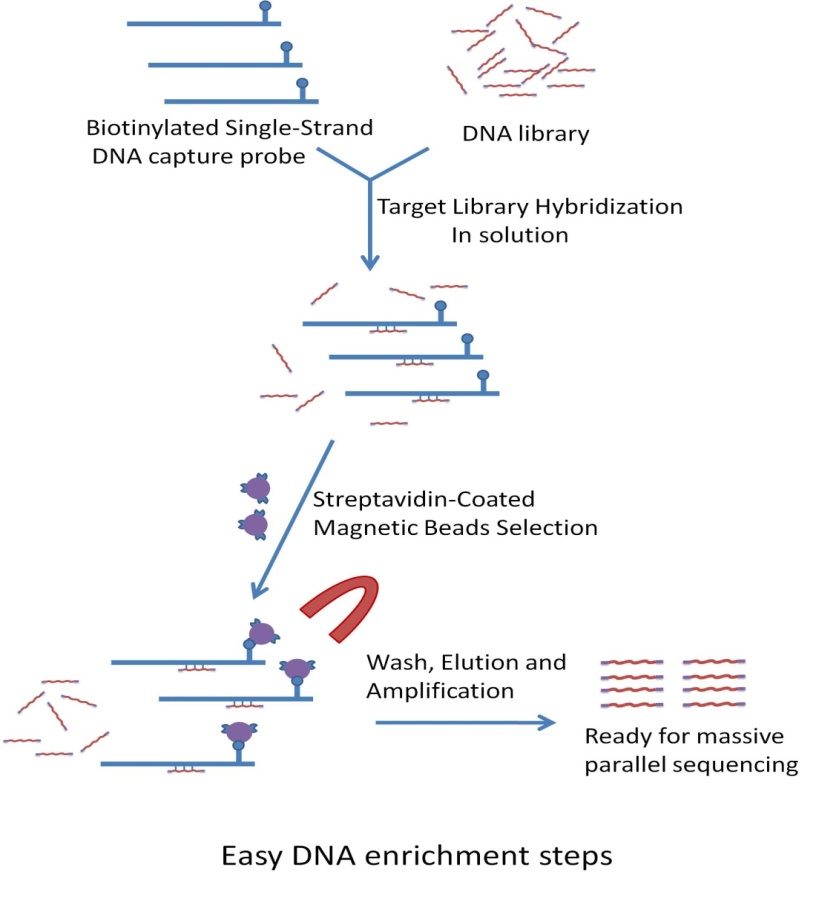
***

**General Scheme of the target region related gene enrichment process**

**Bioinformatics analysis**

For nuclear gene sequencing analysis, high-quality reads were retrieved from raw reads by filtering out the low quality reads and adaptor sequences using the Solexa QA package and the cutadapt program (<http://code.google.com/p/cutadapt/>), respectively. SOAPaligner program was then used to align the clean read sequences to the human reference genome (hg19).

After the PCR duplicates were removed by the Picard software, the SNPs was firstly identified using the SOAPsnp program (<http://soap.genomics.org.cn/soapsnp.html>). Subsequently, we realigned the reads to the reference genome using BWA and identified the insertions or deletions (InDels) using the GATK program (<http://www.broadinstitute.org/gsa/wiki/index.php/Home_Page>). The identified SNPs and InDels were annotated using the Exome-assistant program (<http://122.228.158.106/exomeassistant>). MagicViewer was used to view the short read alignment and validate the candidate SNPs and InDels. Nonsynonymous variants were evaluated by four algorithms, Ployphen, SIFT, PANTHER and Pmut, as described previously to determine pathogenicity.

For mitochondria sequencing data analysis, the low quality reads were filtered using the Trim Galore program. Then, 3'/5' adapters were trimmed using the Cutadapt program implemented in Trim Galore. Only reads with >20 sequencing quality and readlength >80bp were retained. The clean reads were aligned to the reference human genome hg19 using the BWA program. The quality scores were recalibrated and the reads were realigned to hg19 using the GATK software package. SNVs were detected and genotyped using the GATK UnifiedGenotyper in single-sample mode (with parameters -im ALL -mbq 20 -mmq 20 -mm42 3 -deletions 0.05). Variants were filtered with GATK VariantFiltration module (with filters “QUAL<50.0 & QD<5.0 & HRun>10 & DP<4” and parameters –cluster 3 -window 10). Indels were detected with GATKIndelGenotyperV2 (with parameters -im ALL) and filtered with a custom python module that removed sites with amax_cons_av≥1.9 (maximum average number of mismatches across reads supporting the indel) or max_cons_nqs_av_mm ≥0.2 (maximumaverage mismatch rate in the 5-bp NQS window around the indel, across indel-supporting reads). The coverage of each position on the mitochondria genome was plotted along the base position, the plot was seen with a large fragment deletion compared with the normal samples, as shown in figure 1.
