# Supplementary material for: Mutation analysis of Chinese sporadic congenital sideroblastic anemia by targeted capture sequencing
Source: J Hematol Oncol. 2015 May 20;8:55. doi: 10.1186/s13045-015-0154-0 (PMC4490691; doi:10.1186/s13045-015-0154-0)
Supplement: Additional file 4: Figure S2. — Chromatograms of the mutations found in the patients, as confirmed by Sanger sequencing. [file 13045_2015_154_MOESM4_ESM.pdf]

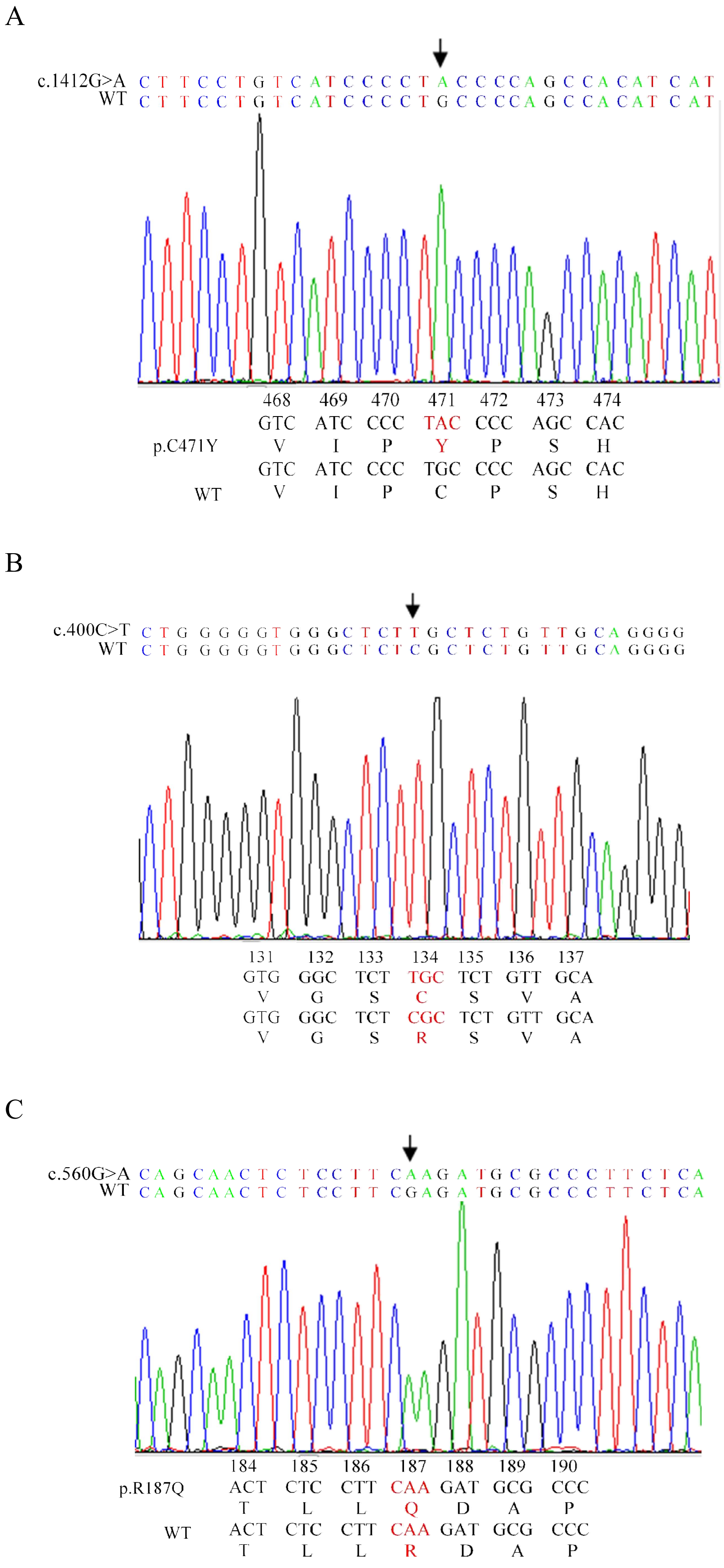
A
c.1412G>A CTTCCTGTCATCCCCTACCCCAGCCACATCAT
WT CTTCCTGTCATCCCCTGCCCCAGCCACATCAT
468 469 470 471 472 473 474
GTC ATC CCC TAC CCC AGC CAC
p.C471Y V I P Y P S H
GTC ATC CCC TGC CCC AGC CAC
WT V I P C P S H
B
c.400C>T CTGGGGGTGGGCTCTTGCTCTGTTGCAGGGG
WT CTGGGGGTGGGCTCTCGCTCTGTTGCAGGGG
131 132 133 134 135 136 137
GTG GGC TCT TGC TCT GTT GCA
V G S C S V A
GTG GGC TCT CGC TCT GTT GCA
V G S R S V A
C
c.560G>A CAGCAACTCTCCTTCAAGATGCGCCCTTCTCA
WT CAGCAACTCTCCTTCGAGATGCGCCCTTCTCA
184 185 186 187 188 189 190
p.R187Q ACT CTC CTT CAA GAT GCG CCC
T L L Q D A P
WT ACT CTC CTT CAA GAT GCG CCC
T L L R D A P

D

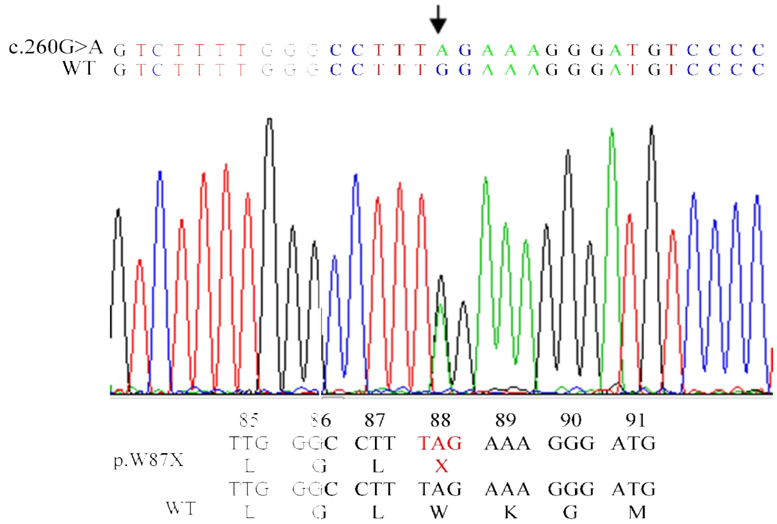

E

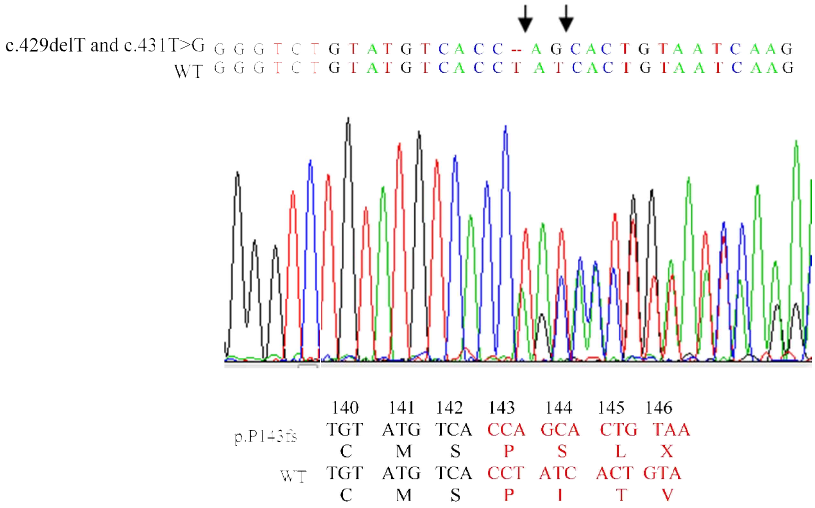

Additional file 4. Chromatograms of the mutations found in the patients, as confirmed by Sanger sequencing. (A) Homozygous missense mutation c.1412G>A of ALAS2 in Patient No. 3. (B) Homozygous missense mutation c.400C> T of SLC25A38 in Patient No. 4. (C) Homozygous mis-sense mutation c.560G>A of SLC25A38 in Patient No. 5. (D) Heterozygous nonsense mutation c.260G>A of SLC25A38 in Patient No. 6. (E) Heterozygous nonsense mutations c.429delT and c.431T>G of SLC25A38 in Patient No. 6.
