# Supplementary material for: Mutation analysis of Chinese sporadic congenital sideroblastic anemia by targeted capture sequencing
Source: J Hematol Oncol. 2015 May 20;8:55. doi: 10.1186/s13045-015-0154-0 (PMC4490691; doi:10.1186/s13045-015-0154-0)
Supplement: Additional file 5: Figure S3 — Potential model of three-dimensional structure of human ALAS2 (A) and SLC25A38 (B) and their evolutionary conservation of the residues with missense mutations. The color scale range from blue to red represent the conservation scores from 1-most variable to 9-most conserved. The color code for residues with missense mutations C471 (A), R134 (B) are 6, 9, respectively. Surface-mapping of phylogenetic information was done with ConSurf; view is from the cytosolic side. [file 13045_2015_154_MOESM5_ESM.pdf]

A

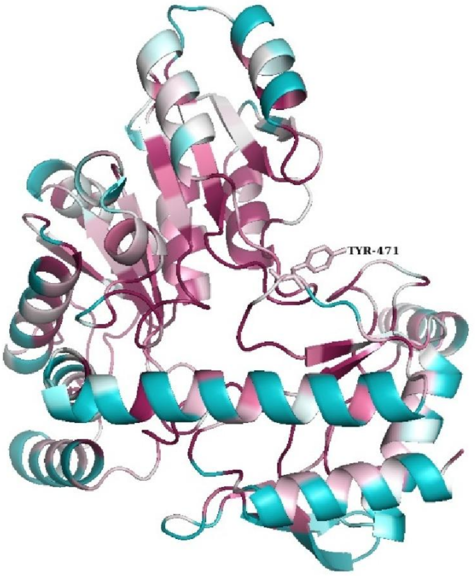

B

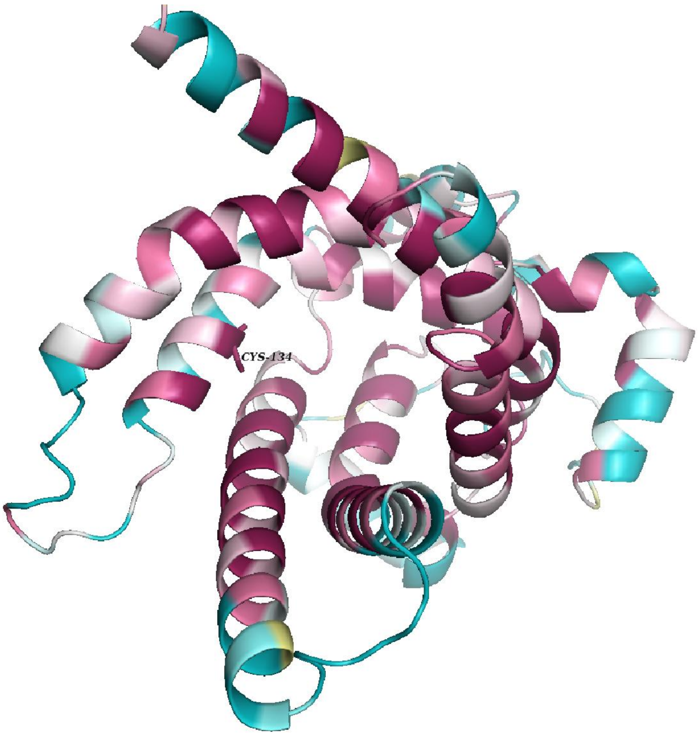

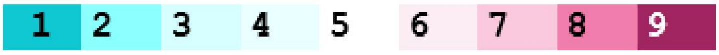

Addtional file 5. Potential model of three-dimensional structure of human ALAS2 (A) and SLC25A38 (B) and their evolutionary conservation of the residues with missense

mutations. The color scale range from blue to red represent the conservation scores from 1-most variable to 9-most conserved. The color code for residues with missense mutations C471 (A), R134 (B) are 6, 9, respectively. Surface-mapping of phylogenetic information was done with ConSurf; view is from the cytosolic side.
